# Treatment strategies, complications, and outcomes in spontaneous cerebellar hemorrhage: a swedish observational single-center study

**DOI:** 10.1007/s00701-026-06872-w

**Published:** 2026-04-15

**Authors:** Hilin Sida, Rozerin Kevci, Fartein Velle, Anders Lewén, Andreas Fahlström, Per Enblad, Teodor Svedung Wettervik

**Affiliations:** Department of Medical Sciences, Section of Neurosurgery, Uppsala University, Uppsala, 751 85 Sweden

**Keywords:** Cerebellar hemorrhage, Clinical decision-making, Neurointensive care, Outcome, Stroke

## Abstract

**Purpose:**

Spontaneous cerebellar hemorrhage (sCH) is associated with high mortality, but favorable outcomes can be achieved with appropriate surgical management. We evaluated treatment strategies, complications, outcomes, and prognostic factors in sCH patients at a tertiary center.

**Methods:**

Adults with primary sCH treated at the neurointensive care unit in Uppsala, Sweden, between 2008 and 2024 were retrospectively included. Clinical and radiological data were collected. Patients were managed conservatively or surgically according to institutional protocols. Outcomes were mortality at discharge and 6 months, and functional outcome at NIC discharge assessed with the Glasgow Outcome Scale-Discharge (GODS). Predictors of 6-month mortality and favorable outcome (GODS > 3) were analyzed.

**Results:**

A total of 194 patients were included; 50% underwent surgery. Surgically treated patients had lower admission Glasgow Coma Scale motor scores, larger hematoma volumes, and more infratentorial mass effect. Among awake patients with hematomas > 15 mL initially managed conservatively, 78% did not require delayed surgery and most achieved favorable outcomes. Combined hematoma evacuation, suboccipital decompression, and external ventricular drainage (EVD) was associated with low complication rates and low early mortality. Selected patients with hydrocephalus and smaller hemorrhages were successfully treated with EVD alone. Overall mortality was 11% at discharge and 28% at 6 months. Age, neurological status, and hematoma volume independently predicted mortality.

**Conclusions:**

Favorable outcomes after sCH are achievable, including in elderly patients. Conservative management is appropriate in neurologically stable patients with moderate hematoma volumes, while EVD alone may suffice in selected cases with isolated hydrocephalus.

**Supplementary Information:**

The online version contains supplementary material available at 10.1007/s00701-026-06872-w.

## Introduction

Spontaneous cerebellar hemorrhage (sCH) constitutes a small subset (≈10%) of intraparenchymal brain bleedings [4] and differs fundamentally from supratentorial intracerebral hemorrhage in many aspects [5]. Due to the restricted volume of the posterior fossa, even relatively small bleedings (≈ > 15 mL) may rapidly lead to brainstem compression, obstructive hydrocephalus, and death [2, 5, 11, 18, 28]. However, if such life-threatening infratentorial mass effect is treated efficiently, the local parenchymal destruction typically results in less debilitating sequelae than in supratentorial hemorrhage [3, 5, 14, 17, 28]. Although level I-evidence is lacking, surgical evacuation of large sCH and external ventricular drainage (EVD) in secondary hydrocephalus are widely adopted practices and are associated with relatively favorable functional outcomes [2, 5, 7, 18, 28]. Accordingly, current guidelines recommend surgical intervention in sCH with brainstem compression, clinical deterioration, acute hydrocephalus, or hematoma diameter > 3 cm/15 mL [5, 21]. However, these recommendations are based largely on heterogeneous retrospective data, subjected to substantial indication bias, and several important areas of uncertainty persist [5, 21].

First, in case of moderately large sCH (≈15 mL) in awake patients without hydrocephalus and only mild mass effect in the posterior fossa, surgical management remains controversial. Particularly, some reports suggest that most of these patients do not deteriorate and recover well without surgery [19], highlighting that expectant management may suffice in many of these cases. Second, EVD alone does not relieve brainstem compression and the risk of upward herniation needs to be considered. However, whether this is a safe approach under certain circumstances, characterized by predominant obstructive hydrocephalus with milder mass effect in the posterior fossa, remain to be explored [6]. Third, the less pronounced sequalae caused by sCH compared to supratentorial intracerebral hemorrhages increases the chance to obtain a favorable outcome after surgery also in higher ages but more knowledge is warranted for better selection criteria. It remains unclear whether there is a chronological or biological threshold beyond which surgical intervention and neurointensive care (NIC) become futile [28], particularly in frail patients receiving antithrombotic therapy that may exacerbate hematoma growth and complicate operative management. Fourth, the optimal surgical strategy, including suboccipital bone decompression with clot evacuation vs. clot evacuation alone vs. minimally invasive approaches [5, 6, 12, 15, 28], and the subsequent NIC management targets [28] are poorly defined. Our institution routinely performs suboccipital bone decompression with hematoma evacuation in combination with EVD followed by NIC, including ICP/CPP management strategies extrapolated from traumatic brain injury (TBI) and subarachnoid hemorrhage [25, 26]. Yet, comprehensive cohort-level data describing outcomes, complications, and predictors of prognosis within such treatment paradigms are scarce.

While selection and indication bias cannot be fully avoided in retrospective research, the present observational study sought to provide a comprehensive cohort-level analysis of sCH at our center by: (1) characterizing clinical and radiological differences between conservatively and surgically managed sCH patients; (2) examining the clinical course and outcomes of initially conservatively treated patients with large hematomas and those treated with EVD alone; (3) assessing decision-making, management, and outcomes in elderly patients, including those on antithrombotic therapy; and (4) evaluating surgical outcomes and predictors within the cohort.

## Materials and methods

### Patients and study design

In this retrospective, observational study, all patients with sCH who had been referred to the NIC unit, Uppsala University Hospital, between 1 January 2008 to 31 August 2024, were eligible for inclusion. The screening population was identified through a comprehensive review of hospital records and administrative databases and comprised all patients admitted to the NIC unit during that time period with any of the diagnostic codes I16.3 and I16.4. Children (age < 18 years) and sCH cases due to secondary etiologies (e.g., hemorrhage due to trauma, intracranial tumors, vascular malformations, or neurosurgical procedures) were excluded. Altogether, 194 sCH patients were included for this study (Supplementary Fig. 1).

### Management protocol

The neurosurgical department at Uppsala University Hospital serves as a tertiary referral center for a catchment area of approximately 2 million inhabitants. Most patients diagnosed with sCH were initially assessed and managed at their local hospital according to general guidelines [5, 21]. The on-call neurosurgeon is in general consulted for essentially all patients diagnosed with intracranial hemorrhages. Regarding patients with sCH, those with moderate-to-large hematoma volumes on the initial or subsequent CT scan, who were not in a severely compromised neurological condition, had limited comorbidity, and were typically younger than 85 years were selected for referral to the NIC unit for observation and/or neurosurgical intervention.

The patients were managed according to our institutional management protocol throughout the study period, as described below and in a previous study [28]. Surgical treatment was considered in patients who were drowsy or unconscious (Glasgow Coma Scale [GCS] score ≤ 13) in combination with radiological signs of significant mass effect, particularly compression of the fourth ventricle and/or brainstem. The final decision regarding indication for surgery as well as choice of surgical strategy was made by the responsible neurosurgeon. The preferred surgical approach at our institution consists of EVD in combination suboccipital bone decompression and evacuation of the sCH. EVD alone was considered sufficient only in patients with minor sCH, but substantial intraventricular hemorrhage (IVH) and acute hydrocephalus. The EVD was inserted into the right frontal horn. Then the patient was turned to prone positioning. A midline skin incision and dissection were made and a wide bilateral suboccipital bone decompression was performed (from below the transverse sinus to the foramen magnum), followed by dural opening and evacuation of the sCH. In selected cases, the posterior arch of the atlas was removed for additional decompression. If the posterior fossa remained relatively constricted, duraplasty was considered and the wound was subsequently closed in multiple layers [28]. In very selected exceptional cases, it was allowed to reinsert the bone flap if a very sufficient internal decompression had been achieved.

Patients were managed in the NIC unit with the focus on optimizing cerebral physiology. Treatment targets included ICP < 20 mmHg, CPP > 60 mmHg, normothermia, normal electrolytes, pO_2_ > 12 kPa, normoventilation, and blood glucose levels maintained between 5 and 10 mM [28]. Neurological wake-up tests were performed 3–6 times daily. Sedation was primarily achieved with propofol, with morphine used for analgesia. If ICP was above 20 mm Hg the EVD was opened and adjusted to achieve a drainage level of 15 mmHg after a CT scan ruled out postoperative mass lesion. Patients obeying commands were extubated. If the patient did not wake up and signs of hydrocephalus remained, CSF was drained at lower levels.

### Data collection – clinical and radiological variables

Data on clinical variables were retrieved from medical records. Demographic variables included age, sex, Charlson Comorbidity Index (CCI) [23], use of antithrombotic agents prior to hemorrhage, and how these agents were handled post-ictus (discontinuation, reversal etc.). Clinical severity was assessed using the GCS Motor score (GCS M) and pupillary reactivity (normal vs one or both pupils unreactive) at presentation in the emergency department (ED), on admission to the NIC unit, and immediately prior to surgery (only in the surgical group).

In the surgical cohort, time from ictus to surgery and from NIC unit admission to surgery were recorded. Surgical strategy was categorized as hematoma evacuation with concomitant EVD, EVD alone, or initial EVD followed by secondary hematoma evacuation. Technical aspects of surgery, including suboccipital craniectomy, laminectomy of C1, and performance of duraplasty, were documented. In addition, duration of EVD monitoring (days), procedure-related complications such as EVD-related meningitis or reoperation due to residual hematoma/postoperative hematoma, need for extension of bony decompression, or CSF leakage, and the need for permanent cerebrospinal fluid diversion (ventriculoperitoneal [VP] shunt) were registered.

Radiological variables included hematoma laterality (uni-/bilateral), brainstem hemorrhage, hematoma volume, signs of posterior fossa mass effect, and secondary hydrocephalus. Hematoma volume was calculated using the ABC/2 method [29]. Brainstem compression was graded as none, minimal, moderate, or severe [2]. Fourth ventricle compression was categorized as none, minimal, moderate, or severe obliteration [10, 28]. Tonsillar herniation was defined as descent of the cerebellar tonsils below the foramen magnum (yes/no). The extent of IVH was quantified using the Graeb score [11], and ventricular enlargement was assessed using Evans’ index (frontal horn width/maximal biparietal diameter) [27]. All radiological measures were evaluated on admission CT, preoperatively in surgically treated patients and at maximal radiological progression. All radiological assessments were performed by one of the authors (R.K.).

### Outcome

Mortality/survival was assessed at three time points; at NIC discharge, 6 months post-ictus, and at any time point during follow-up (only used in the Kaplan–Meier analyses). Functional outcome was only possible to assess at NIC discharge, using the Glasgow Outcome Scale at Discharge (GODS) [13], ranging from 1 (death) to 5 (good recovery). Favorable outcome was defined as GODS > 3.

### Statistical analysis

The statistical analyses were conducted in RStudio software (version 4.4.2) [1]. Categorical variables were presented as numbers (percentages) and ordinal/continuous variables as medians (interquartile range [IQR]). Differences in demographic, clinical, and radiological variables between the surgical (sCH evacuation and/or EVD) and conservative treatment groups were evaluated with Mann–Whitney U- or Chi-square-test, depending on the type of data. The patients with moderate-to-large sCH volume (> 15 mL on the first CT), in good clinical condition (GCS M = 6), who were managed conservatively during the first 24 h, were studied in particular in relation to hematoma progression, the need for delayed surgery, and outcome at discharge.

The surgical sCH cohort was also described in detail in regards to preoperative clinical and radiological variables, surgical aspects, and complications. These variables were assessed in relation to the surgical strategy (sCH evacuation + EVD vs EVD—> sCH evacuation vs EVD alone), while we avoided statistical tests due to the limited number of patients in some cohorts.

The association between demographic, clinical, radiological, and management related variables in relation to favorable outcome at discharge and mortality at 6 months was evaluated using univariable logistic regressions. In multivariable analyses, variables were selected a priori to reflect key and complementary aspects of the underlying pathophysiology. Specifically, we included age as the main demographic variable, admission GCS M as a measure of clinical grade, and three radiological variables: sCH volume as a marker of cerebellar tissue destruction, brainstem compression as a measure of mass effect, and the Evans index as an indicator of secondary hydrocephalus. In addition, one further variable showing the strongest associations with outcome in univariable analyses was included, depending on what was statistically permissible given the number of outcome events for each multivariable regression model, in order to avoid overfitting. Odds ratios (ORs) and their corresponding 95% confidence intervals (CIs) were reported. Survival probabilities were estimated using the Kaplan–Meier method, with follow-up time defined in months from ictus to last follow-up date, and death as the event of interest. Patients were stratified into age groups (≤ 49, 50–59, 60–69, 70–79, ≥ 80 years). Differences in survival between age strata were assessed using the log-rank test. Kaplan–Meier curves were plotted with corresponding risk tables using the *survival()* and *survminer()* packages. A p-value below 0.05 was considered statistically significant.

## Results

### Demography, admission status, and radiological variables – entire cohort

In the total cohort of 194 sCH patients (Table 1), the median age was 67 years (IQR 60–73), with a male/female distribution of 62/38%. The median CCI was 0 (IQR 0–1) and 40% were treated with antithrombotic agents, equally divided between antiplatelets and anticoagulants. Essentially all antithrombotic agents were discontinued and anticoagulants, in particular, were also reversed (Supplementary Table 1). Most patients had a GCS M score of 6 at both ED presentation and NIC admission. Median sCH volume on first CT was 19 mL (IQR 11–30), with brainstem hemorrhage in 6% (Table 2). Median Graeb score was 2 (IQR 0–4) and median Evans’ index 0.30 (IQR 0.27–0.33) on the first CT. Brainstem compression was seen in 15%, moderate-to-severe fourth ventricular compression in 59%, and tonsillar herniation in 8%. These radiological features showed slight deterioration on maximal/worst CT. The number of surgically and conservatively managed patients was equal (*n* = 97 in each sub-group).

**Table 1:** Demography, admission status, and outcome—comparison between surgically and conservatively managed patients

Variables	Entire cohort	Conservative group	Surgical group	*P*-value
*Demography*				
Patients, *n* (%)	194 (100%)	97 (50%)	97 (50%)	NA
Age (years), median (IQR)	67 (60–73)	66 (60–74)	68 (60–73)	0.862
Sex (male/female), *n* (%)	120/74 (62/38%)	62/35 (64/36%)	58/39 (60/40%)	0.657
CCI (scale), median (IQR)	0 (0–1)	0 (0–2)	0 (0–1)	**0.046**
Antithrombotic agents				0.279
None, *n* (%)	117 (60%)	57 (59%)	60 (62%)	
Antiplatelets, *n* (%)	33 (17%)	18 (19%)	15 (16%)	
Anticoagulants, *n* (%)	35 (18%)	15 (16%)	20 (21%)	
Both antiplatelets and anticoagulants, *n* (%)	9 (5%)	7 (7%)	2 (2%)	
*Neurological function*				
GCS M at the ED (scale), median (IQR)	6 (6–6)	6 (6–6)	6 (6–6)	0.957
GCS M at NIC admission (scale), median (IQR)	6 (4–6)	6 (6–6)	6 (4–6)	**0.010**
GCS M before surgery, median (IQR)	5 (4–6)	NA	5 (4–6)	NA
Pupillary status at the ED (abnormal), *n* (%)	13 (7%)	9 (9%)	4 (4%)	0.251
Pupillary status at NIC admission (abnormal), *n* (%)	21 (11%)	15 (16%)	6 (6%)	0.065
Pupillary status before surgery (abnormal), *n* (%)	6 (6%)	NA	6 (6%)	NA
Favorable outcome at NIC discharge, *n* (%)	65 (34%)	42 (43%)	23 (23%)	**0.006**
Mortality at NIC discharge, *n* (%)	22 (11%)	17 (18%)	5 (5%)	**0.013**
Mortality at 6-months, *n* (%)	55 (28%)	27 (28%)	28 (29%)	1.000

Bold indicates statistical significance (*p* < 0.05)

Abnormal pupillary status was defined as one or two unreactive pupils

In the conservatively managed group, post-discharge mortality after 6 months occurred among 10 patients (10%), where the majority of deaths was found in patients with a GODS score ≤ 3 at NIC discharge (9 patients)

In the surgically managed group, mortality increased from 5% at NIC discharge to 29% at the 6-month follow-up. Among the 23 patients post-discharge mortality after 6 months, 20 patients had GODS score ≤ 3 at NIC discharge

*CCI* Charlson comorbidity index, *ED* Emergency department, *GCS M* Glasgow Coma Scale Motor score, *GODS* Glasgow Outcome Scale at Discharge, *IQR* Interquartile range, *NA* Not applicable, *NIC* Neurointensive care

**Table 2:** Radiological variables—comparison between surgically and conservatively managed patients

	Entire cohort	Conservative group	Surgical group	*P*-value
Localization (uni-/bilateral), *n* (%)	143/51 (74/26%)	73/24 (75/25%)	70/27 (72/28%)	0.744
Brainstem involvement (yes), *n* (%)	12 (6%)	1 (1%)	11 (11%)	**0.007**
Hematoma volume on first CT (mL), median (IQR)	19 (11–30)	15 (6–25)	22 (15–33)	**0.001**
Maximal hematoma volume (mL), median (IQR)	20 (12–33)	17 (7–28)	25 (16–37)	** < 0.001**
Hematoma volume (mL) before surgery, median (IQR)	25 (16–38)	NA	25 (16–378)	NA
Brainstem compression on first CT (none/minimal/moderate/severe), *n* (%)	164/23/7/0 (85/12/4/0%)	88/8/1/0 (91/8/1/0%)	76/15/6/0 (78/16/6/0%)	**0.037**
Maximal brainstem compression (none/minimal/moderate/severe), *n* (%)	159/24/11/0 (82/12/6/0%)	86/7/4/0 (89/7/4/0%)	73/17/7/0 (75/18/7/0%)	**0.049**
Brainstem compression (none/minimal/moderate/severe) before surgery, *n* (%)	72/18/7/0 (74/19/7/0%)	NA	72/18/7/0 (74/19/7/0%)	NA
Compression of the 4th ventricle (none/minimal/moderate/severe) on first CT, *n* (%)	54/81/50/9 (28/42/26/5%)	40/34/19/4 (41/35/20/4%)	14/47/31/5 (14/49/32/5%)	**0.001**
Maximal compression of the 4th ventricle (none/minimal/moderate/severe), *n* (%)	42/73/60/19 (22/38/31/10%)	35/33/21/8 (36/34/22/8%)	7/40/39/11 (7/41/40/11%)	** < 0.001**
Compression of the 4th ventricle (none/minimal/moderate/severe) before surgery, *n* (%)	6/42/39/10 (6/43/40/10%)	NA	6/42/39/10 (6/43/40/10%)	NA
Tonsil herniation (yes) on first CT, *n* (%)	15 (8%)	5 (5%)	10 (10%)	0.282
Maximal tonsil herniation (yes), *n* (%)	20 (10%)	7 (7%)	13 (13%)	0.238
Tonsil herniation (yes) before surgery, *n* (%)	13 (13%)	NA	13 (13%)	NA
Graeb score (scale) on first CT, median (IQR)	2 (0–4)	0 (0–2)	2 (1–6)	** < 0.001**
Maximal Graeb score (scale), median (IQR)	2 (0–5)	1 (0–2)	3 (1–7)	** < 0.001**
Graeb score (scale) before surgery, median (IQR)	3 (1–6)	NA	3 (1–6)	NA
Evans’ index on first CT (ratio), median (IQR)	0.30 (0.27–0.33)	0.29 (0.27–0.32)	0.31 (0.28–0.34)	**0.008**
Maximal Evans’ index (ratio), median (IQR)	0.32 (0.28–0.35)	0.30 (0.28–0.33)	0.34 (0.30–0.36)	** < 0.001**
Evans’ index (ratio) before surgery, median (IQR)	0.32 (0.28–0.34)	NA	0.32 (0.28–0.34)	NA

Bold indicates statistical significance (*p* < 0.05)

*CT* Computed tomography, *IQR* Interquartile range, *NA* Not applicable

### Demography, admission status, and radiological variables – comparison between conservatively and surgically managed patients

While age, sex, and usage of antithrombotic agents did not differ between treatment groups, surgically managed patients had slightly lower CCI (*p* = 0.046) and GCS M scores at NIC admission (*p* = 0.010). They also exhibited larger sCH volumes (*p* = 0.001), more frequent brainstem hemorrhage (*p* = 0.007) and compression (*p* = 0.037), greater fourth ventricular compression (*p* = 0.001), higher Graeb scores (*p* < 0.001), and higher Evans’ index (*p* = 0.008) on initial CT.

### Clinical course in initially conservatively treated sCH patients with moderate-to-large bleeding volume

**Fig. 1:**
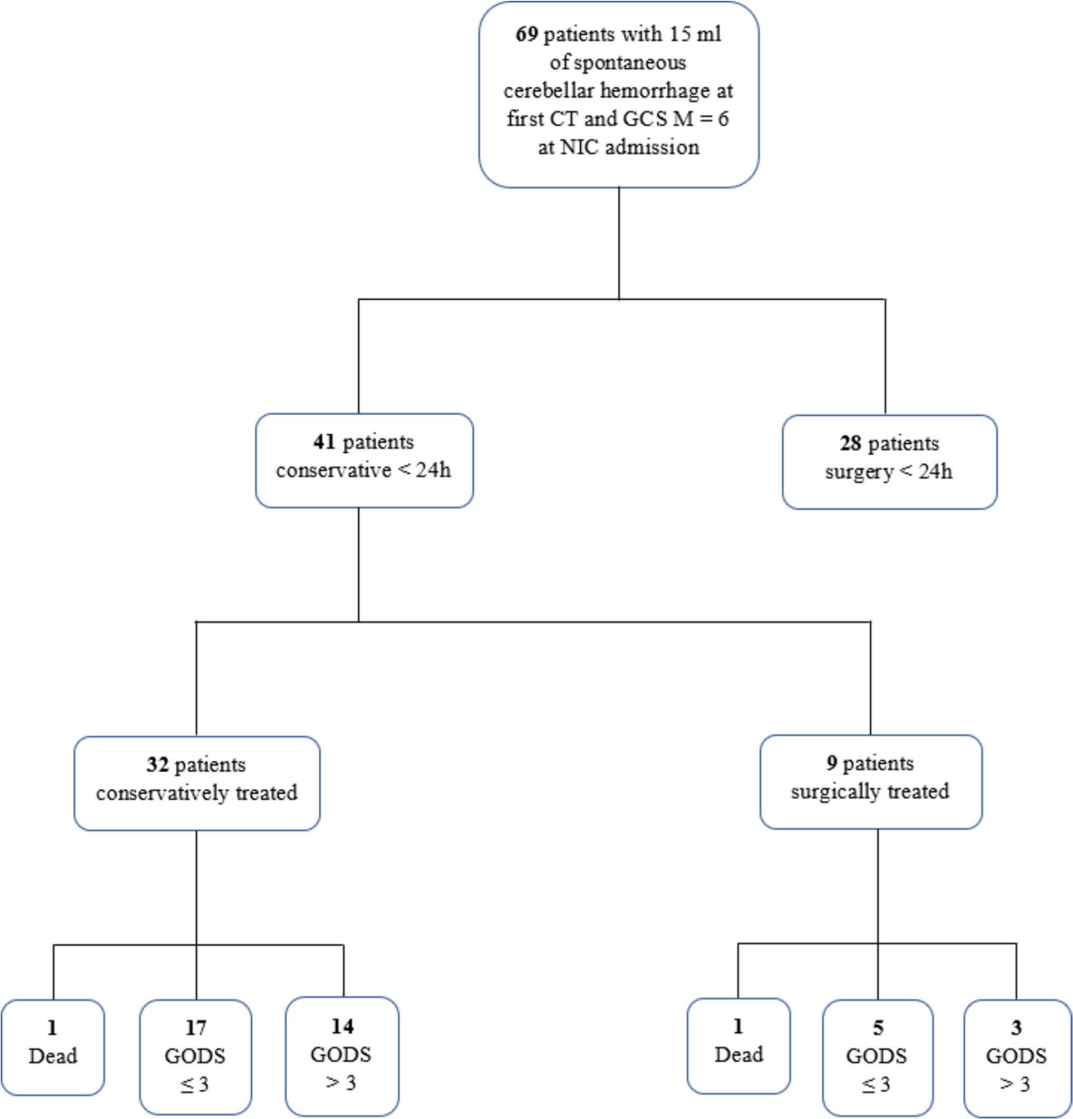
Management dendrogram of patients with moderate-to-large sCH. The dendrogram is based on all patients with sCH of 15 mL or larger on the first CT. Of 41 patients with such large bleeding volumes, who were GCS M = 6 at NIC admission and conservatively managed during the first 24 h, only 9 were treated with delayed sCH evacuation. The surgically treated patients exhibited clinical deterioration, with a clear preoperative decline in level of consciousness. Radiological progression was also observed, including a slight increase in lesion volume and Evans’s index, a slightly higher Graeb scores, as well as a greater compression of the 4th ventricle and basal cisterns. CT = Computed tomography. GCS M = Glasgow Coma Scale Motor score. GODS = Glasgow Outcome Scale at Discharge. NIC = Neurointensive care. sCH = Spontaneous cerebellar hemorrhage

As illustrated in Fig. 1, there were 69 patients with a 15 mL sCH volume at the first CT and GCS M 6 at NIC admission, of whom 41 (59%) were managed conservatively the first 24 h. Of these 41 patients, 9 (22%) required surgery and 3 (33%) of them recovered favorably. Among the remaining 32 patients who were managed conservatively, 1 (3%) died during NIC while 14 (44%) recovered favorably.

### Surgical treatments – indication, timing, technical aspects, and complications

In the surgical group (Table 3), the median GCS M decreased from 6 (IQR 6–6) at ED presentation to 5 (IQR 4–6) preoperatively, while the rate of abnormal pupillary reactivity remained stable at ~ 5%. Median time to surgery was 10 h (IQR 6–15) from ictus and 4 h (IQR 2–8) from NIC admission.

**Table 3:** Timing, surgical aspects, and complications in the surgical cohort

Variables	Surgical cohort	Surgical evacuation + EVD	EVD alone	EVD—> Surgical evacuation
Patients, *n* (%)	97 (100%)	71 (73%)	22 (23%)	4 (4%)
*Timing*				
Time from ictus to surgery (hours), median (IQR)	10 (6–15)	9 (6–14)	12 (2–14)	24 (15–32)
Time from NIC admission to surgery, median (IQR)	4 (2–8)	4 (1–7)	7 (3–8)	9 (6–12)
*Surgical aspects*				
Evacuation of hemorrhage (yes), *n* (%)	75 (77%)	71 (100%)	0 (0%)	4 (100%)
Suboccipital craniectomy (yes), *n* (%)	65 (67%)	61 (86%)	0 (0%)	4 (100%)
C1 laminectomy (yes), *n* (%)	44 (45%)	42 (59%)	0 (0%)	2 (50%)
Duraplasty (yes), *n* (%)	49 (51%)	45 (63%)	1 (5%)	3 (75%)
Duration with EVD (days), median (IQR)	7 (4–12)	7 (5–12)	7 (3–11)	5 (3–6)
*Complications*				
Reoperation due to postoperative hematoma (yes), *n* (%)	2 (2%)	2 (3%)	0 (0%)	0 (0%)
Enlargement of bony decompression (yes), *n* (%)	2 (2%)	2 (3%)	0 (0%)	0 (0%)
Reoperation due to CSF leakage (yes), *n* (%)	2 (2%)	2 (3%)	0 (0%)	0 (0%)
Culture-verified EVD-related meningitis (yes), *n* (%)	7 (7%)	5 (7%)	2 (9%)	0 (0%)
*Chronic impairment of CSF circulation*				
VP-shunt (yes), *n* (%)	8 (8%)	8 (11%)	0 (0%)	0 (0%)

*CSF* Cerebrospinal fluid, *EVD* External ventricular drainage, *IQR* Interquartile range, *NIC* Neurointensive care, *VP* Ventriculo-peritoneal

Three surgical treatment pathways were identified. Most patients underwent (i) immediate combined sCH evacuation and EVD insertion (*n* = 71 [73%]). Twenty-six patients (27%) received initial EVD placement, of whom 85% (ii) did not require further surgical procedures while 15% were later operated with sCH removal (iii). Suboccipital craniectomy was performed in essentially all cases undergoing sCH evacuation; C1 laminectomy and duraplasty were performed in approximately 60%.

As shown in Supplementary Table 2, patients treated with immediate sCH evacuation and EVD had larger hematomas (median 29 mL [IQR 20–38]) compared with EVD alone (10 mL [IQR 6–17]) and EVD followed by evacuation (23 mL [IQR 13–35 mL]). Median Graeb score was slightly higher in the EVD-only group (3 vs 2 and 1, respectively). Preoperative Evans’ index was similar across groups (0.30–0.32).

Reoperation for postoperative or residual hematoma was required in 2/75 patients (3%); rates of extended bony decompression and CSF leakage were similarly low in the cohort treated with both sCH evacuation and EVD (< 5%). Culture-verified EVD-related meningitis occurred in 7%. Ultimately, 8% in the entire surgical cohort required VP shunt placement for chronic hydrocephalus.

### Clinical outcome

At discharge, overall mortality was 11% and favorable outcome 34% (Table 1). Mortality at discharge was higher in conservatively managed patients (18% vs 5%, *p* = 0.013), whereas favorable outcome at discharge was more frequent (43% vs 23%, *p* = 0.006). At 6 months, mortality was similar between groups, just below 30% (*p* = 1.000).

### Predictive factors of mortality and favorable outcome

In univariable analyses of mortality at 6 months (Table 4), higher age, usage of antithrombotic agents pre-ictus, higher CCI, lower admission GCS M, abnormal pupillary reactivity, larger sCH volume, brainstem compression, fourth ventricle compression, higher Graeb score, and higher Evans’ index were associated with increased mortality (all *p* ≤ 0.050). In multivariable models, higher age (*p* < 0.001), lower NIC admission GCS M (*p* < 0.001), and larger sCH volume (*p* = 0.003) remained independently associated with mortality. For favorable outcome, univariable analyses showed associations with higher NIC admission GCS M, smaller sCH volume, absence of brainstem or fourth ventricle compression, lower Graeb score, and lower Evans’ index (all *p* < 0.01). In multivariable analyses, higher NIC admission GCS M (*p* = 0.006) and smaller sCH volume (*p* = 0.027) remained independently associated with favorable outcome.

**Table 4:** Predictive factors of mortality and favorable outcome – uni- and multivariable analyses

Variables	Mortality				Favorable outcome			
	Univariable		Multivariable		Univariable		Multivariable	
	OR (95% CI)	*p*	OR (95% CI)	*p*	OR (95% CI)	*p*	OR (95% CI)	*p*
*Demography*								
Age (years)	1.05 (1.02–1.08)	**0.005**	1.09 (10.4–1.14)	** < 0.001**	1.00 (0.98–1.02)	0.982	1.01 (0.98–1.05)	0.4
Sex (female)	1.00 (0.52–1.90)	0.995	NA	NA	1.21 (0.61–2.07)	0.706	NA	NA
Antithrombotic agents (yes)	1.90 (1.01–3.60)	**0.046**	NA	NA	0.69 (0.37–1.27)	0.239	NA	NA
CCI (scale)	1.44 (1.10–1.90)	**0.008**	NA	NA	0.81 (0.60–1.08)	0.168	NA	NA
*Clinical severity*								
GCS M at NIC admission	0.51 (0.41–0.63)	** < 0.001**	0.46 (0.33–0.60)	** < 0.001**	3.34 (1.97–7.44)	** < 0.001**	2.44 (1.47–5.49)	**0.006**
Pupillary status at NIC admission (abnormal)	10.99 (4.03–35.37)	** < 0.001**	NA	NA	0.30 (0.07–0.93)	0.060	NA	NA
*Radiological variables*								
Localization (bilateral)	1.56 (0.78–3.09)	0.202	NA	NA	0.78 (0.38–1.53)	0.471	NA	NA
Hematoma volume on first CT (mL)	1.07 (1.05–1.10)	** < 0.001**	1.05 (1.02–1.08)	**0.003**	0.94 (0.91–0.97)	** < 0.001**	0.97 (0.93–1.00)	**0.027**
Brainstem compression on first CT (yes)	5.15 (2.30–11.93)	** < 0.001**	1.01 (0.31–3.15)	> 0.9	0.05 (0.00–0.26)	**0.005**	0.21 (0.01–1.32)	0.2
Compression of the 4th ventricle on first CT (severe)	10.05 (3.46–42.71)	** < 0.001**	NA	NA	0.33 (0.17–0.64)	** < 0.001**	NA	NA
Graeb score on first CT (scale)	1.18 (1.08–1.31)	** < 0.001**	NA	NA	0.80 (0.69–0.89)	** < 0.001**	0.93 (0.80–1.07)	0.3
Evans’ index on first CT (ratio; per 0.1)	2.42 (1.10–5.48)	**0.029**	0.72 (0.23–2.19)	0.9	0.27 (0.11–0.59)	**0.001**	0.50 (0.19–1.28)	0.2

Mortality = mortality at 6 months post-ictus

Favorable outcome = GODS > 3 at NIC unit discharge

Bold indicates statistical significance (*p* < 0.05)

Mortality: Nagelkerke = 0.49 (*n* = 194); AUROC (95% CI) = 0.88 (0.83–0.93)

Favorable outcome: Nagelkerke = 0.36 (*n* = 194); AUROC (95% CI) = 0.80 (0.74–0.86)

*AUROC* Area under the receiver operating characteristic curve, *CCI* Charlson comorbidity index, *CT* Computed tomography, *GCS M* Glasgow Coma Scale Motor score, *GODS* Glasgow Outcome Scale at Discharge, *NA* Not applicable, *NIC* Neurointensive care

**Fig. 2:**
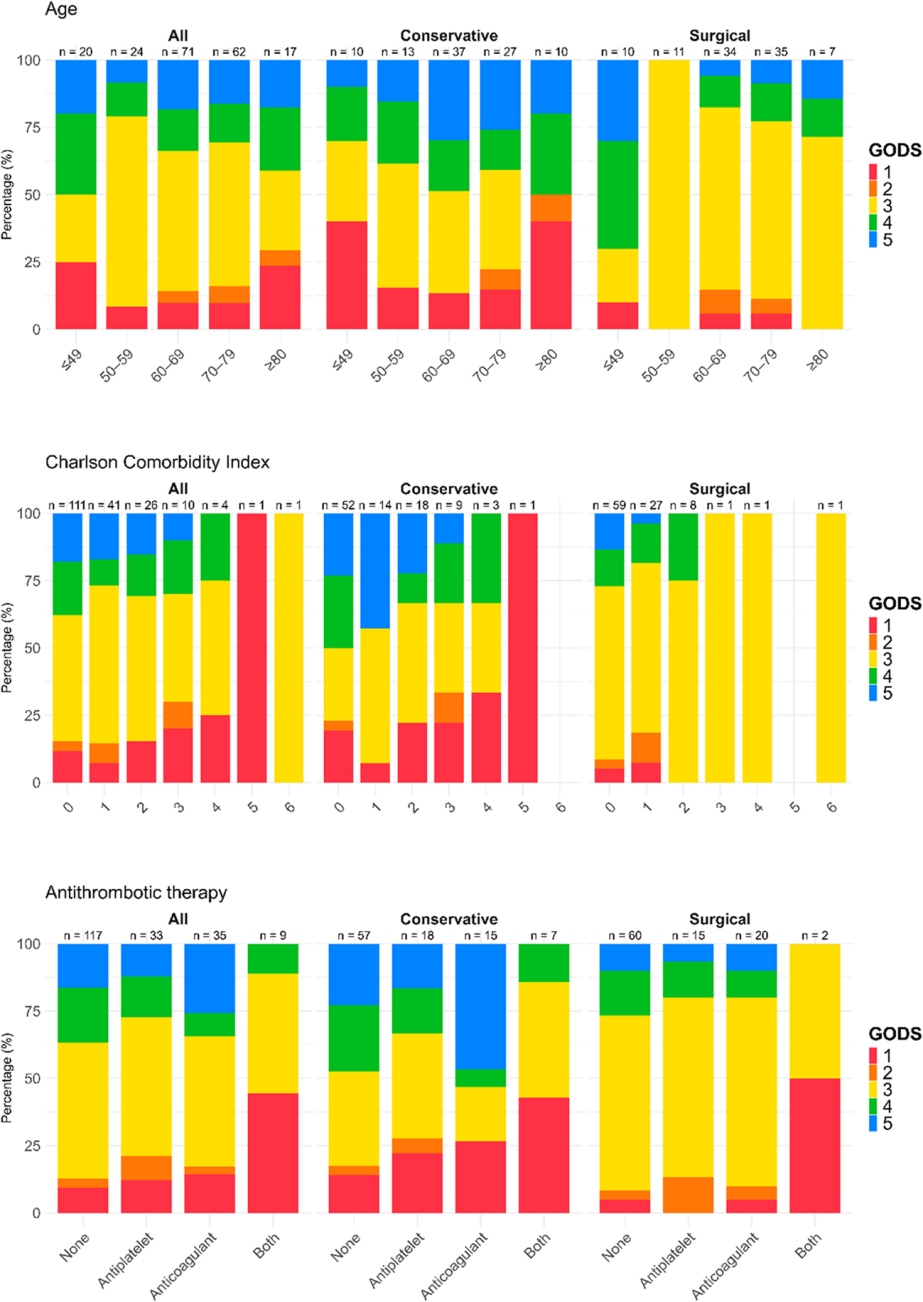
GODS in relation to age, co-morbidity, and antithrombotic agents in the entire cohort and in sub-cohorts managed conservatively and surgically. The figures illustrate the outcome distribution in relation to age, Charlson Co-morbidity Index, and usage of antithrombotic agents pre-ictus in the entire cohort and in the sub-cohorts treated conservatively and with surgery, respectively. GODS = Glasgow Outcome Scale at Discharge

**Fig. 3:**
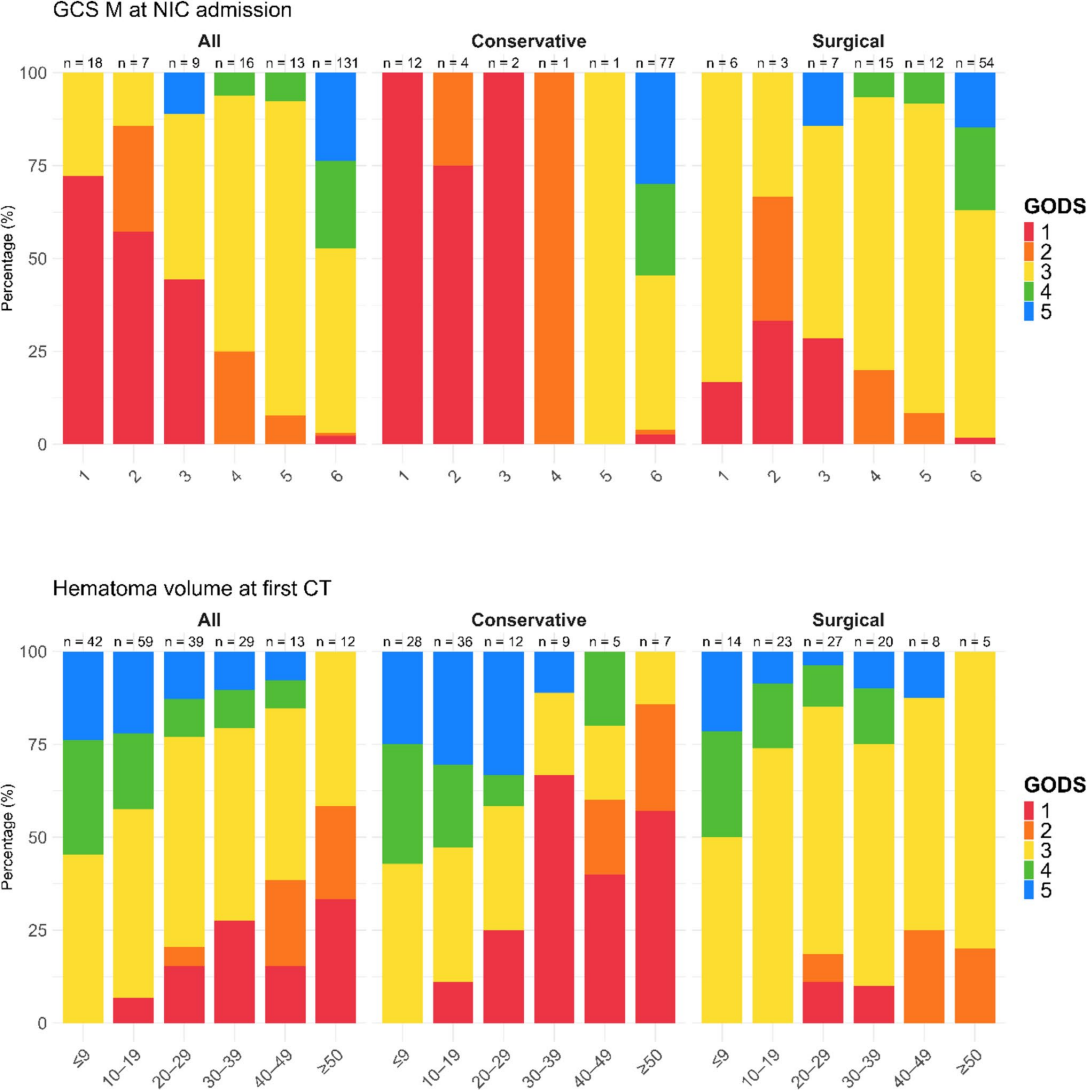
GODS in relation to clinical severity (GCS M at NIC admission) and imaging characteristics (Hematoma volume on first CT) in the entire cohort and in sub-cohorts managed conservatively and surgically. The figures illustrate the outcome distribution in relation to GCS M at NIC admission and sCH volume in the entire cohort and in the sub-cohorts treated conservatively and with surgery, respectively. CT = Computed tomography. GCS M = Glasgow Coma Scale Motor score. GODS = Glasgow Outcome Scale at Discharge. NIC = Neurointensive care. sCH = Spontaneous cerebellar hemorrhage

Figure 2 and 3 also visualize the association between GODS and age categories, CCI, antithrombotic therapy, GCS M at NIC admission, and sCH volume at first CT in the conservatively managed and surgical group. Notably, in the conservatively managed group, mortality was close to 100% for patients GCS M < 4 and no patient exhibited a favorable outcome for GCS M at NIC admission < 6. In the surgical cohort, favorable outcome was also most common for GCS M at 6, but was also found for lower categories down to 3 and mortality was still below 30% for GCS M < 4. In addition, mortality increased and the rate of favorable outcome decreased with higher sCH volumes, this was most pronounced for bleedings above 30 mL in the conservative group. Furthermore, Supplementary Fig. 2 visualizes the outcome distribution for each surgical treatment strategy.

**Fig. 4:**
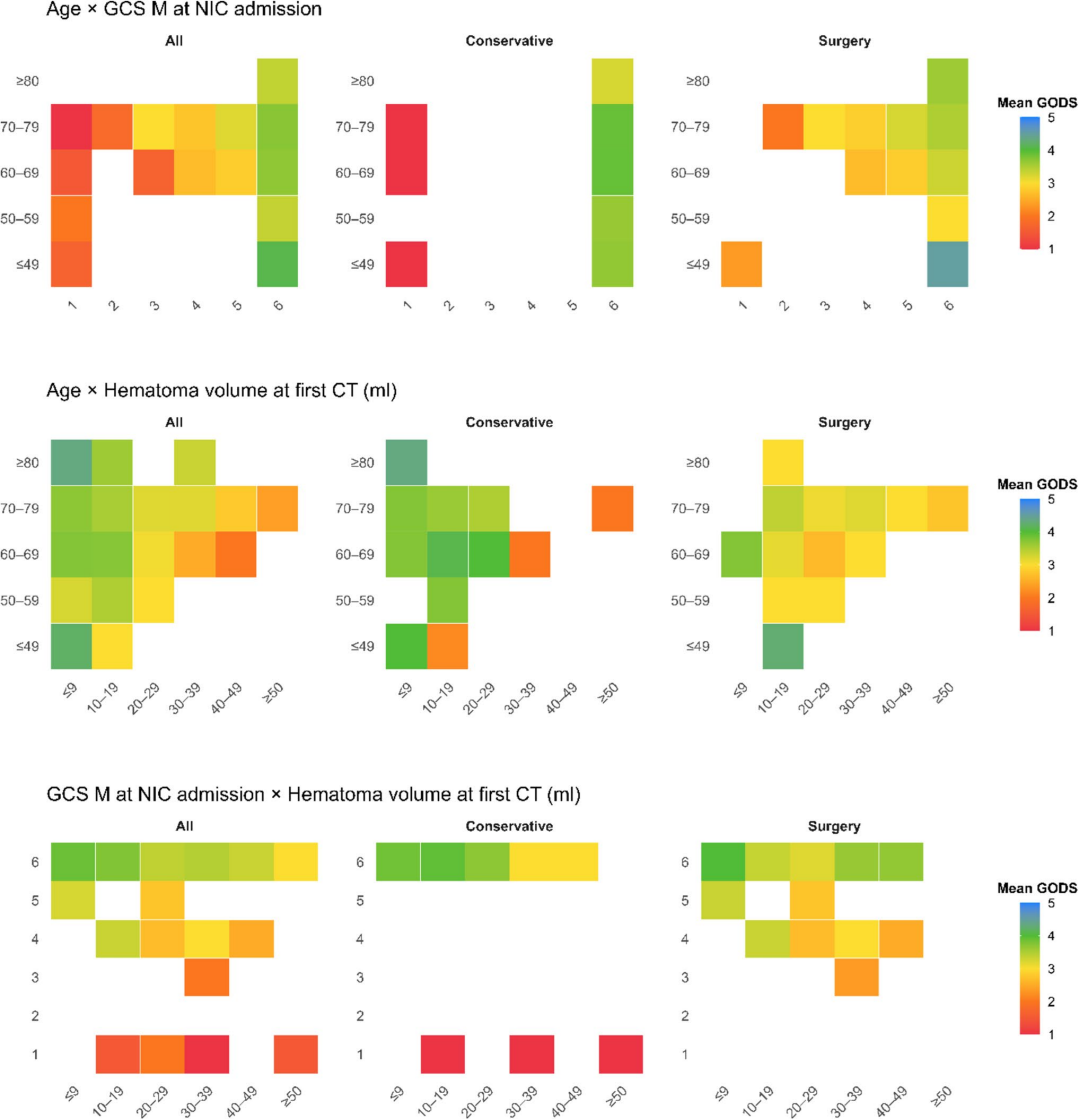
Combination plots of clinical and radiological characteristics in relation to GODS. These plots illustrate the color-coded mean GODS value for certain combinations of clinical and radiological characteristics. The grid cells were colored as white if they included less than three patients. CT = Computed tomography. GCS M = Glasgow Coma Scale Motor score. GODS = Glasgow Outcome Scale at Discharge. NIC = Neurointensive care. sCH = Spontaneous cerebellar hemorrhage

**Fig. 5:**
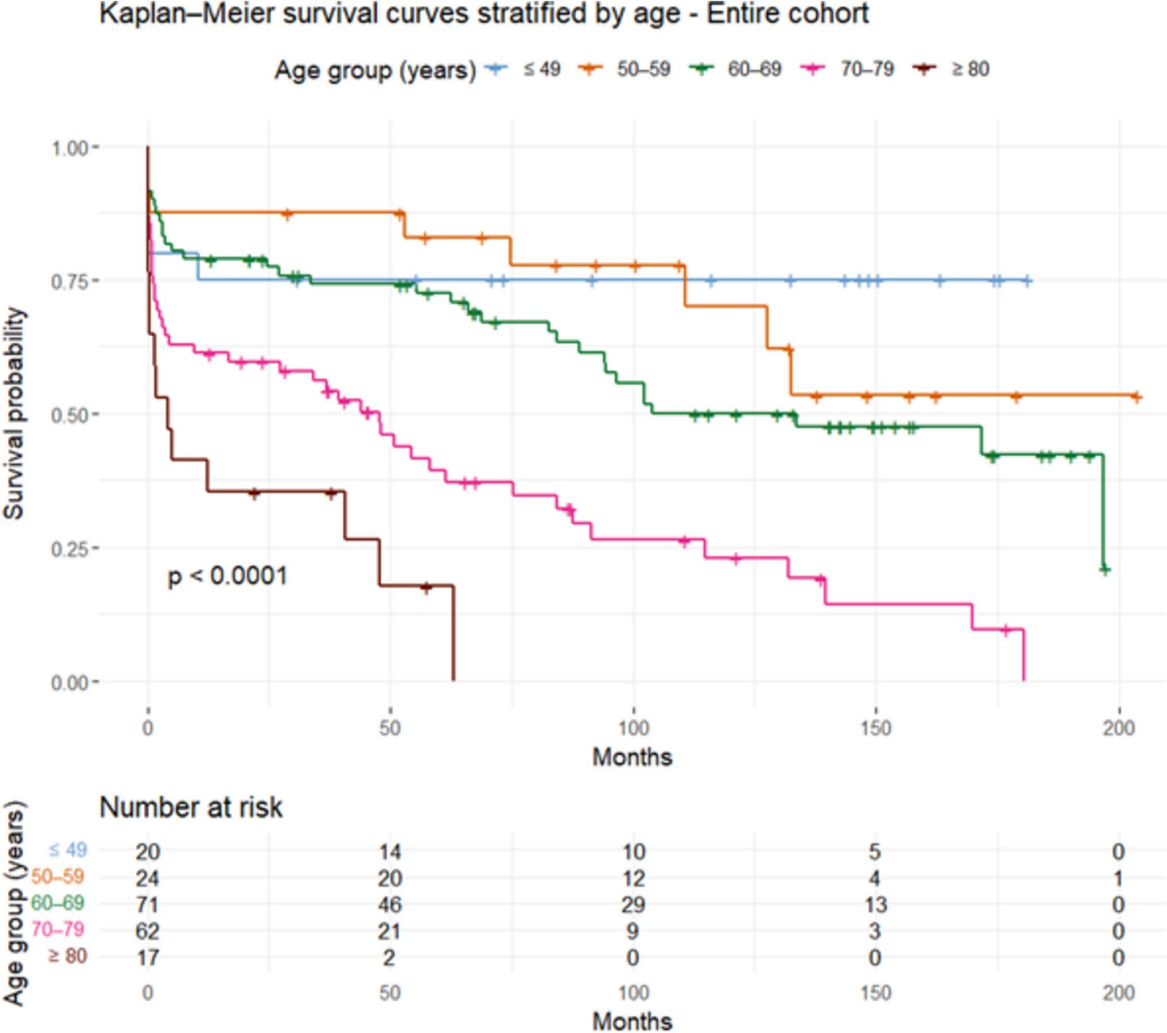
Long-term mortality in relation to age – a Kaplan–Meier analysis. *p* < 0.05 indicates statistical significance

In Kaplan–Meier analysis of mortality (Fig. 4 and Supplementary Fig. 3), it was illustrated that the greatest drop in mortality occurred within the first six months after ictus across all age groups. Notably, all 80 + patients in the surgical cohort were deceased after approximately 3 years and all cases after 5 years post-ictus in the conservative group for the same age group. In the younger cohorts, the mortality rate was lower, but continued also after the first 6–12 months, e.g., around 50% were deceased both in the 50–59 and 60–69 years within 10 years post-ictus (Fig. 5).

## Discussion

In this 17-year NIC cohort comprising 194 patients with sCH, we addressed several key clinical dilemmas in contemporary sCH management. Our findings indicate that a substantial proportion of patients with moderately large hematomas and good neurological status can be safely managed conservatively, while a smaller subset may deteriorate and still benefit from timely surgical intervention followed by NIC. The data further support the safety of EVD-only management in carefully selected patients with predominant obstructive hydrocephalus, as well as the feasibility of emergency neurosurgery and NIC care in elderly patients, including those receiving antithrombotic therapy. With a relatively liberal admission strategy, approximately half of the patients ultimately required surgical treatment, primarily those with greater clinical and radiological severity. Despite this, early mortality was low and favorable functional outcomes were achieved in a considerable proportion of patients, including selected octogenarians. Overall, outcome was predominantly determined by the combined burden of age, neurological status, and radiological injury severity.

### Patient cohort, management of antithrombotic agents, and decision-making – conservative vs. surgical approach

Primary sCH is most commonly related to cerebral microvascular disease, e.g., following chronic arterial hypertension, and predominantly affects elderly patients [5, 16]. Accordingly, the majority of patients in our cohort was aged ≥ 60 years, and nearly half was receiving antithrombotic therapy at ictus. At a population level, this reflects a relatively frail patient group with somewhat limited remaining life expectancy. Nevertheless, despite advanced age, the overall comorbidity burden was modest, with CCI scores typically ranging from 0 to 1, likely reflecting a degree of selection toward patients considered suitable for active neurosurgical management. Still, several patients aged > 80 years were admitted for potential neurosurgical management, reflecting both the aging general population affected by such conditions and the expectation among the responsible clinicians that favorable outcomes may still be achievable even at advanced age. Antithrombotic therapy was managed according to current guidelines [5, 21], with discontinuation at presentation in virtually all cases and those receiving anticoagulants were treated with appropriate reversal agents. Importantly, the use of antithrombotic agents did not differ between conservatively and surgically managed patients, indicating that surgical intervention was not withheld due to bleeding risk alone. However, more aggressive reversal strategies were employed in the surgical subgroup, including adjunctive use of desmopressin, platelet transfusions, and more frequent administration of tranexamic acid. Importantly, despite the common use of antithrombotic agents in this cohort, the reoperation rate due to postoperative bleeding was low (< 5%).

The equal distribution between conservative and surgical management reflects an institutional strategy of early admission of sCH patients at risk of rapid deterioration. The conservative cohort mostly consisted of patients with moderate but relatively smaller sCH volumes who were actively monitored, but never deteriorated. In contrast, surgically treated patients were more severely affected at admission or later on, with lower GCS M, larger sCH volumes, greater mass effect in the posterior fossa, more IVH, and hydrocephalus. Most surgically treated hemorrhages were large (≈30 mL), frequently compressing the 4th ventricle and accompanied obstructive hydrocephalus, findings that are expected, uncontroversial, and largely consistent with surgical indication in current guidelines [5, 21]. A small part of the conservative group also consisted of these severe cases, but they deteriorated too much in clinical and radiological grade, often during interhospital transfer, and surgical management was not considered meaningful in those cases.

However, the more challenging clinical dilemma concerns patients with moderate-to-large hemorrhages (> 15 mL) who initially appear neurologically stable (GCS M = 6). In our cohort, around 80% of such patients managed conservatively during the first 24 h did not require surgical intervention and a great proportion of these cases had recovered favorably at discharge. Moreover, 3 cases in the subset of 9 patients who deteriorated and were treated surgically had a favorable outcome at discharge. Still, surgically treated patients may require longer rehabilitation, and it is possible that outcomes at 6 months would have been even more favorable. Nevertheless, on a group level, patients initially managed conservatively demonstrated promising overall outcomes.

### Surgical approach – hematoma evacuation, suboccipital decompression and EVD vs. EVD alone

The vast majority of surgically treated patients underwent sCH evacuation combined with suboccipital decompression and EVD insertion, typically performed immediately upon admission to the NIC unit. These patients represented the most severe end of the disease spectrum, with large hematoma volumes, 4th ventricle compression, mass effect in the posterior fossa, IVH, and hydrocephalus. Despite the aggressive nature of this approach, the complication rate was low, with reoperations due to bleeding, enlargement of bony decompression, or CSF leakage required in < 5% of cases, while mortality was around 5% at discharge. These findings further support that the surgical approach, evacuation of sCH combined with suboccipital decompression (and often duraplasty) and EVD, is safe and effective. This finding is consistent with previous studies on the benefit of sCH evacuation with suboccipital bone decompression [6, 28]. Although bone flap replacement or minimally invasive techniques are inherently less aggressive, the morbidity associated with suboccipital bone decompression in this setting appears low and provides contribute to the relief of the posterior fossa, which may be critical given the risk of postoperative swelling in the posterior fossa. Nevertheless, future studies are needed to benchmark these approaches and to delineate scenarios in which more or less invasive surgical strategies are most appropriate.

A selected subgroup was managed with EVD alone, primarily in patients with relatively small sCH volumes (≈10 mL) but pronounced IVH and ventriculomegaly. Despite deviating from current guideline recommendations [5, 21], this strategy appeared safe and was associated with favorable short-term outcomes (Supplementary Fig. 2). Four patients required delayed hematoma evacuation; these had larger initial hematoma volumes (median 23 mL), placing them closer to the surgical threshold at presentation. Importantly, outcomes remained acceptable despite delayed intervention. Thus, while guidelines emphasize upfront evacuation in neurologically impaired patients or those with moderate-to-large sCH, our findings suggest that EVD alone may be sufficient in carefully selected cases with predominant hydrocephalus and no mass effect.

### Short- and long-term outcomes – age, neurological grade, and bleeding volume – who will recover?

In the entire sCH cohort, short-term mortality was surprisingly low at discharge at 11%, with much lower rate in surgically treated patients compared with conservatively managed patients (5% vs 18%), the latter partly reflecting patients deemed too ill or neurologically compromised to undergo surgery. Notably; by six months, 28% of patients had died, with similar mortality rates in the surgical and conservative groups, indicating that a substantial proportion of early survivors (but low GODS), particularly in the surgical cohort, did not successfully transition through step-down care and rehabilitation. However, mortality did not stop during the recovery period during the first 6–12 months. As illustrated in the Kaplan–Meier plot, long-term mortality was substantial and continued relatively rapidly with around 50% deaths over the subsequent 10 years also in relatively younger patients between 50 to 70 years. Our findings appear consistent with a recent detailed report on long-term outcomes in sCH patients, with similar findings who also discovered that sCH patients tend to exhibit a substantial burden of repeated cardiovascular events including ischemic and hemorrhagic stroke, and myocardial infarction [16]. We believe that further studies are warranted to explore whether sCH patients could have long-term benefits from secondary cardiovascular preventive investigations and treatments.

However, the excess mortality was most pronounced among patients aged ≥ 80 years, all of whom died within 5 (only 3 in the surgical cohort) years after ictus. This observation should be interpreted in the context of background life expectancy; in Sweden, life expectancy has been close to 80 years during this period [24]. Thus, patients aged ≥ 80 years were already close to, or beyond, the population-average expected lifespan, and survival for several additional years after an event as severe as sCH may represent a clinically meaningful outcome rather than treatment failure. In the perspective of the old patients, the gain of a few more years with acceptable quality of life may be of tremendous value. The clinical course should also be compared to other neurosurgical conditions in the elderly, such as glioblastoma, where survival despite active multimodal therapy is often measured in months [20].

In a recent systematic review, mortality at discharge was reported to be approximately 25–30% in both conservatively and surgically treated patients, increasing to around 40% at longer-term follow-up in surgically treated cohorts [18]. These figures were slightly lower in our cohort but overall largely comparable. Comparison of favorable functional outcome across studies remains challenging due to substantial heterogeneity in outcome measures (e.g., modified Rankin Scale versus GODS and other scales), definitions of favorable outcome, and timing of assessment, ranging from discharge to several years post-ictus [2, 6, 11, 18, 28]. Reported rates of favorable long-term outcome generally vary between 20 and 40%, but interpretation is limited by the lack of consistency in these methodological factors [2, 6, 11, 18, 28]. Our results may also be interpreted in relation to the ICH score [8]. Given that all patients had infratentorial hemorrhage, the majority presented with an associated IVH, many had reduced consciousness (GCS < 13), and a certain proportion had sCH volumes exceeding 30 mL (although relatively few were > 80 years), most patients would have an estimated ICH score of 2–4. This corresponds to a predicted 30-day mortality exceeding 20% (for ICH score 2) and substantially higher at scores ≥ 3 [8]. In this context, the observed mortality in our cohort appears relatively favorable.

In analyses of prognostic factors, higher age was, as expected, independently and significantly associated with 6-month mortality. While age is an intuitive and robust risk factor, the clinically relevant question in treatment decision-making is whether surgical intervention offers a reasonable chance of benefit, rather than being futile or even harmful in terms of suffering. Our data suggest that a substantial proportion of elderly patients, including selected octogenarians, may still derive meaningful benefit from active treatment. Still, age must be taken into account as a prognostic factor in combination with other important variables when it comes to decision-making, which needs to be individualized and multifactorial.

Other significant prognostic variables included neurological status, with a lower GCS M score being independently associated with poorer outcome. As illustrated in Fig. 3, the transition toward unfavorable outcomes occurred primarily at GCS M < 6. In conservatively managed patients, GCS M < 6 was almost uniformly fatal, whereas surgical intervention appeared to mitigate this risk: even patients with GCS M scores of 3–4 occasionally regained consciousness by discharge. Although the probability of favorable short-term outcome was markedly reduced at these levels, recovery was not precluded. Radiological markers of injury severity, including larger sCH volume, mass effect in the posterior fossa, IVH, and hydrocephalus, were, as expected, associated with increased mortality and lower rates of favorable outcome. In the conservatively managed cohort, hematoma volumes exceeding 30 mL were generally associated with very poor prognosis.

Lastly, outcome appeared to depend on co-existing prognostic variables. As illustrated in the combination plots (Fig. 4), certain constellations, such as advanced age combined with low GCS M, or low GCS M combined with large hematoma volume, were particularly unfavorable compared with the converse scenarios. Altogether, in line with other acute brain injury conditions, age, neurological grade, and radiological markers of lesion severity carried substantial explanatory value for outcome [9, 22].

### Methodological considerations

This study has several notable strengths. First, it comprises a relatively large cohort of patients with sCH, with detailed and systematically collected clinical and radiological data. Second, although the analyses were retrospective, patient management followed a prospectively implemented institutional protocol [28], ensuring consistency in admission criteria, monitoring, and treatment decisions over time.

Several limitations should be acknowledged. First, the present work primarily focused on surgical decision-making and interventions. Optimization of cerebral physiology during NIC, an aspect likely to influence outcome, was not addressed in detail and will be the focus of future investigations. Second, while overall mortality could be assessed with long-term follow-up of up to 17 years, functional outcome was limited to discharge status (GODS). Functional recovery may evolve beyond hospital discharge, and more prolonged follow-up would likely capture additional improvements in patients who survive the early post-injury phase and undergo active rehabilitation. Third, this is a single-center study, reflecting local demographics, referral patterns, NIC logistics, and rehabilitation pathways. Although this allows for internally consistent analyses, external validity may be limited. Evaluation of treatment strategies in other settings, within the Nordic countries, Europe, and beyond, would be valuable.

## Conclusions

Most patients with primary sCH were elderly and almost half received antithrombotic agents before ictus. In sCH patients with bleedings larger than 15 mL, who were still awake, initial conservative management was often successful. Still, 22% of these patients later required surgery, but most of them with good outcomes. Otherwise, combined sCH evacuation with suboccipital decompression and EVD was safe and effective with low complication rates and short-term mortality. In cases of hydrocephalus without dominant infratentorial mass effect, EVD alone was a safe and effective alternative and this approach should be considered in selected cases in future guidelines. Short-term outcomes were favorable with low early mortality, but long-term mortality remained substantial and continued over several years, even in relatively younger patients aged 50–70 years. Age, neurological status (GCS M), and sCH volume were the dominant prognostic factors.

## Supplementary Information

Below is the link to the electronic supplementary material.

ESM 1 Supplementary Material 1 (DOCX 179 KB)

ESM 2 Supplementary Material 2 (DOCX 20.1 KB)

## Data Availability

Data are not available due to legal restrictions.

## References

[1] R Core Team (2023) R: A language and environment for statistical computing. R Foundation for Statistical Computing, Vienna, Austria. Available at: https://www.R-project.org/

[2] Dammann P, Asgari S, Bassiouni H, Gasser T, Panagiotopoulos V, Gizewski ER, Stolke D, Sure U, Sandalcioglu IE (2011) Spontaneous cerebellar hemorrhage–experience with 57 surgically treated patients and review of the literature. Neurosurg Rev 34:77–86. 10.1007/s10143-010-0279-0 20697766 10.1007/s10143-010-0279-0

[3] de Oliveira Manoel AL (2020) Surgery for spontaneous intracerebral hemorrhage. Crit Care 24:45. 10.1186/s13054-020-2749-2 32033578 10.1186/s13054-020-2749-2 PMC7006102

[4] Flaherty ML, Woo D, Haverbusch M, Sekar P, Khoury J, Sauerbeck L, Moomaw CJ, Schneider A, Kissela B, Kleindorfer D, Broderick JP (2005) Racial variations in location and risk of intracerebral hemorrhage. Stroke 36:934–937. 10.1161/01.Str.0000160756.72109.95 15790947 10.1161/01.STR.0000160756.72109.95

[5] Greenberg SM, Ziai WC, Cordonnier C, Dowlatshahi D, Francis B, Goldstein JN, Hemphill JC, Johnson R, Keigher KM, Mack WJ, Mocco J, Newton EJ, Ruff IM, Sansing LH, Schulman S, Selim MH, Sheth KN, Sprigg N, Sunnerhagen KS (2022) 2022 guideline for the management of patients with spontaneous intracerebral hemorrhage: a guideline from the American Heart Association/American Stroke Association. Stroke 53:e282. 10.1161/str.0000000000000407 35579034 10.1161/STR.0000000000000407

[6] Hackenberg KA, Unterberg AW, Jung CS, Bösel J, Schönenberger S, Zweckberger K (2017) Does suboccipital decompression and evacuation of intraparenchymal hematoma improve neurological outcome in patients with spontaneous cerebellar hemorrhage? Clin Neurol Neurosurg 155:22–29. 10.1016/j.clineuro.2017.01.019 28226284 10.1016/j.clineuro.2017.01.019

[7] Han J, Lee HK, Cho TG, Moon JG, Kim CH (2015) Management and outcome of spontaneous cerebellar hemorrhage. J Cerebrovasc Endovasc Neurosurg 17:185–193. 10.7461/jcen.2015.17.3.185 26523254 10.7461/jcen.2015.17.3.185 PMC4626341

[8] Hemphill JC, Bonovich DC, Besmertis L, Manley GT, Johnston SC (2001) The ICH score: a simple, reliable grading scale for intracerebral hemorrhage. Stroke 32:891–897. 10.1161/01.str.32.4.891 11283388 10.1161/01.str.32.4.891

[9] Jaja BNR, Saposnik G, Lingsma HF, Macdonald E, Thorpe KE, Mamdani M, Steyerberg EW, Molyneux A, Manoel ALO, Schatlo B, Hanggi D, Hasan D, Wong GKC, Etminan N, Fukuda H, Torner J, Schaller KL, Suarez JI, Stienen MN, Vergouwen MDI, Rinkel GJE, Spears J, Cusimano MD, Todd M, Le Roux P, Kirkpatrick P, Pickard J, van den Bergh WM, Murray G, Johnston SC, Yamagata S, Mayer S, Schweizer TA, Macdonald RL (2018) Development and validation of outcome prediction models for aneurysmal subarachnoid haemorrhage: the SAHIT multinational cohort study. BMJ 360:j5745. 10.1136/bmj.j5745 29348138 10.1136/bmj.j5745

[10] Kirollos RW, Tyagi AK, Ross SA, van Hille PT, Marks PV (2001) Management of spontaneous cerebellar hematomas: a prospective treatment protocol. Neurosurgery 49(6):1378–1386. 10.1097/00006123-200112000-00015 11846937 10.1097/00006123-200112000-00015

[11] Kuramatsu JB, Biffi A, Gerner ST, Sembill JA, Sprügel MI, Leasure A, Sansing L, Matouk C, Falcone GJ, Endres M, Haeusler KG, Sobesky J, Schurig J, Zweynert S, Bauer M, Vajkoczy P, Ringleb PA, Purrucker J, Rizos T, Volkmann J, Müllges W, Kraft P, Schubert AL, Erbguth F, Nueckel M, Schellinger PD, Glahn J, Knappe UJ, Fink GR, Dohmen C, Stetefeld H, Fisse AL, Minnerup J, Hagemann G, Rakers F, Reichmann H, Schneider H, Rahmig J, Ludolph AC, Stösser S, Neugebauer H, Röther J, Michels P, Schwarz M, Reimann G, Bäzner H, Schwert H, Claßen J, Michalski D, Grau A, Palm F, Urbanek C, Wöhrle JC, Alshammari F, Horn M, Bahner D, Witte OW, Günther A, Hamann GF, Hagen M, Roeder SS, Lücking H, Dörfler A, Testai FD, Woo D, Schwab S, Sheth KN, Huttner HB (2019) Association of surgical hematoma evacuation vs conservative treatment with functional outcome in patients with cerebellar intracerebral hemorrhage. JAMA 322:1392–1403. 10.1001/jama.2019.13014 31593272 10.1001/jama.2019.13014 PMC6784768

[12] Li L, Li Z, Li Y, Su R, Wang B, Gao L, Yang Y, Xu F, Zhang X, Tian Q, Zhang X, Guo Q, Chang T, Luo T, Qu Y (2018) Surgical evacuation of spontaneous cerebellar hemorrhage: comparison of safety and efficacy of suboccipital craniotomy, stereotactic aspiration, and thrombolysis and endoscopic surgery. World Neurosurg 117:e90–e98. 10.1016/j.wneu.2018.05.170 29864571 10.1016/j.wneu.2018.05.170

[13] McMillan TM, Weir CJ, Ireland A, Stewart E (2013) The glasgow outcome at discharge scale: an inpatient assessment of disability after brain injury. J Neurotrauma 30:970–974. 10.1089/neu.2012.2703 23230909 10.1089/neu.2012.2703

[14] Mendelow AD, Gregson BA, Fernandes HM, Murray GD, Teasdale GM, Hope DT, Karimi A, Shaw MD, Barer DH (2005) Early surgery versus initial conservative treatment in patients with spontaneous supratentorial intracerebral haematomas in the International Surgical Trial in Intracerebral Haemorrhage (STICH): a randomised trial. Lancet 365:387–397. 10.1016/s0140-6736(05)17826-x 15680453 10.1016/S0140-6736(05)17826-X

[15] Nonaka M, Yagi K, Abe H, Miki K, Morishita T, Iwaasa M, Inoue T (2018) Endoscopic surgery via a combined frontal and suboccipital approach for cerebellar hemorrhage. Surg Neurol Int 9:68. 10.4103/sni.sni_346_17 29721347 10.4103/sni.sni_346_17 PMC5909094

[16] Senff JR, Singh SD, Pasi M, Jolink WMT, Rodrigues MA, Schreuder F, Staals J, Schreuder T, Douwes JPJ, Talsma J, McKaig BN, Kourkoulis C, Yechoor N, Anderson CD, Puy L, Cordonnier C, Wermer MJH, Rothwell PM, Rosand J, Klijn CJM, Al-Shahi Salman R, Rinkel GJE, Viswanathan A, Goldstein JN, Brouwers HB (2024) Long-term outcomes in patients with spontaneous cerebellar hemorrhage: an international cohort study. Stroke 55:1210–1217. 10.1161/strokeaha.123.044622 38487876 10.1161/STROKEAHA.123.044622 PMC11045548

[17] Shu J, Wang W, Ye R, Zhou Y, Tong J, Li X, Lv X, Zhang G, Xu F, Zhang J (2024) Risk factors of prognosis for spontaneous cerebellar hemorrhage: a systematic review and meta-analysis. Acta Neurochir (Wien) 166:291. 10.1007/s00701-024-06174-z 38985355 10.1007/s00701-024-06174-z PMC11236867

[18] Singh SD, Brouwers HB, Senff JR, Pasi M, Goldstein J, Viswanathan A, Klijn CJM, Rinkel GJE (2020) Haematoma evacuation in cerebellar intracerebral haemorrhage: systematic review. J Neurol Neurosurg Psychiatry 91:82–87. 10.1136/jnnp-2019-321461 31848229 10.1136/jnnp-2019-321461

[19] Singh SD, Schreuder F, van Nieuwenhuizen KM, Jolink WM, Senff JR, Goldstein JN, Boogaarts J, Klijn CJM, Rinkel GJE, Brouwers HB (2021) Secondary hematoma evacuation and outcome after initial conservative approach for patients with cerebellar hematoma larger than 3 cm. Neurocrit Care 35(3):680–686. 10.1007/s12028-021-01203-6 33650011 10.1007/s12028-021-01203-6 PMC8692294

[20] Stadler C, Gramatzki D, Le Rhun E, Hottinger AF, Hundsberger T, Roelcke U, Läubli H, Hofer S, Seystahl K, Wirsching HG, Weller M, Roth P (2024) Glioblastoma in the oldest old: clinical characteristics, therapy, and outcome in patients aged 80 years and older. Neuro-Oncol Pract 11:132–141. 10.1093/nop/npad070 10.1093/nop/npad070 PMC10940826 38496908

[21] Steiner T, Purrucker JC, Aguiar de Sousa D, Apostolaki-Hansson T, Beck J, Christensen H, Cordonnier C, Downer MB, Eilertsen H, Gartly R, Gerner ST, Ho L, Holt Jahr S, Klijn CJ, Martinez-Majander N, Orav K, Petersson J, Raabe A, Sandset EC, Schreuder FH, Seiffge D, Al-Shahi Salman R (2025) European stroke organisation (ESO) and European association of neurosurgical societies (EANS) guideline on stroke due to spontaneous intracerebral haemorrhage. Eur Stroke J. 10.1177/23969873251340815 40401775 10.1177/23969873251340815 PMC12098356

[22] Steyerberg EW, Mushkudiani N, Perel P, Butcher I, Lu J, McHugh GS, Murray GD, Marmarou A, Roberts I, Habbema JD, Maas AI (2008) Predicting outcome after traumatic brain injury: development and international validation of prognostic scores based on admission characteristics. PLoS Med 5:e165. 10.1371/journal.pmed.0050165 18684008 10.1371/journal.pmed.0050165 PMC2494563

[23] Sundararajan V, Henderson T, Perry C, Muggivan A, Quan H, Ghali WA (2004) New ICD-10 version of the Charlson comorbidity index predicted in-hospital mortality. J Clin Epidemiol 57:1288–1294. 10.1016/j.jclinepi.2004.03.012 15617955 10.1016/j.jclinepi.2004.03.012

[24] Sundberg L, Agahi N, Fritzell J, Fors S (2018) Why is the gender gap in life expectancy decreasing? The impact of age- and cause-specific mortality in Sweden 1997–2014. Int J Public Health 63:673–681. 10.1007/s00038-018-1097-3 29654335 10.1007/s00038-018-1097-3 PMC6015620

[25] Svedung Wettervik T, Lewén A, Enblad P (2023) Fine tuning of neurointensive care in aneurysmal subarachnoid hemorrhage: from one-size-fits-all towards individualized care. World Neurosurg X 18:100160. 10.1016/j.wnsx.2023.100160 36818739 10.1016/j.wnsx.2023.100160 PMC9932216

[26] Svedung Wettervik TM, Lewén A, Enblad P (2021) Fine tuning of traumatic brain injury management in neurointensive care-indicative observations and future perspectives. Front Neurol 12:638132. 10.3389/fneur.2021.638132 33716941 10.3389/fneur.2021.638132 PMC7943830

[27] Toma AK, Holl E, Kitchen ND, Watkins LD (2011) Evans’ index revisited: the need for an alternative in normal pressure hydrocephalus. Neurosurgery 68:939–944. 10.1227/NEU.0b013e318208f5e0 21221031 10.1227/NEU.0b013e318208f5e0

[28] Tsitsopoulos PP, Tobieson L, Enblad P, Marklund N (2012) Prognostic factors and long-term outcome following surgical treatment of 76 patients with spontaneous cerebellar haematoma. Acta Neurochir (Wien) 154:1189–1195. 10.1007/s00701-012-1372-7 22619023 10.1007/s00701-012-1372-7

[29] Webb AJ, Ullman NL, Morgan TC, Muschelli J, Kornbluth J, Awad IA, Mayo S, Rosenblum M, Ziai W, Zuccarrello M, Aldrich F, John S, Harnof S, Lopez G, Broaddus WC, Wijman C, Vespa P, Bullock R, Haines SJ, Cruz-Flores S, Tuhrim S, Hill MD, Narayan R, Hanley DF (2015) Accuracy of the ABC/2 score for intracerebral hemorrhage: systematic review and analysis of MISTIE, CLEAR-IVH, and CLEAR III. Stroke 46:2470–2476. 10.1161/strokeaha.114.007343 26243227 10.1161/STROKEAHA.114.007343 PMC4550520

