# Supplementary material for: Treatment strategies, complications, and outcomes in spontaneous cerebellar hemorrhage: a swedish observational single-center study
Source: Acta Neurochir (Wien). 2026 Apr 15;168(1):99. doi: 10.1007/s00701-026-06872-w (PMC13086794; doi:10.1007/s00701-026-06872-w)
Supplement: Supplementary file 1 — Supplementary Material 1 (DOCX 179 KB) [file 701_2026_6872_MOESM1_ESM.docx]

**Supplementary Figure 1. Flowchart of inclusion and exclusion of patients.**

**269** patients with spontaneous cerebellar hemorrhage treated at the NIC unit

**75** patients excluded:

63 Due to secondary etiology

11 Due to missing data

1 <18 years

**194** patients included

There were 269 patients with sCH (diagnostic codes I16.3 and I16.4) treated at the NIC unit, Uppsala University Hospital, between 1 January 2008 and 31 August 2024, who were eligible for inclusion in this study. For the 269 patients, 75 were excluded; 63 patients had secondary etiology (e.g., hemorrhage due to trauma, intracranial tumors, vascular malformations, or neurosurgical procedures), 11 patients had missing clinical and radiological data, and 1 patient was younger than 18 years of age. The final cohort included 194 sCH patients.

NIC = Neurointensive care. sCH = Spontaneous cerebellar hemorrhage.

**Supplementary Figure 2. GODS** **in relation to surgical approach**


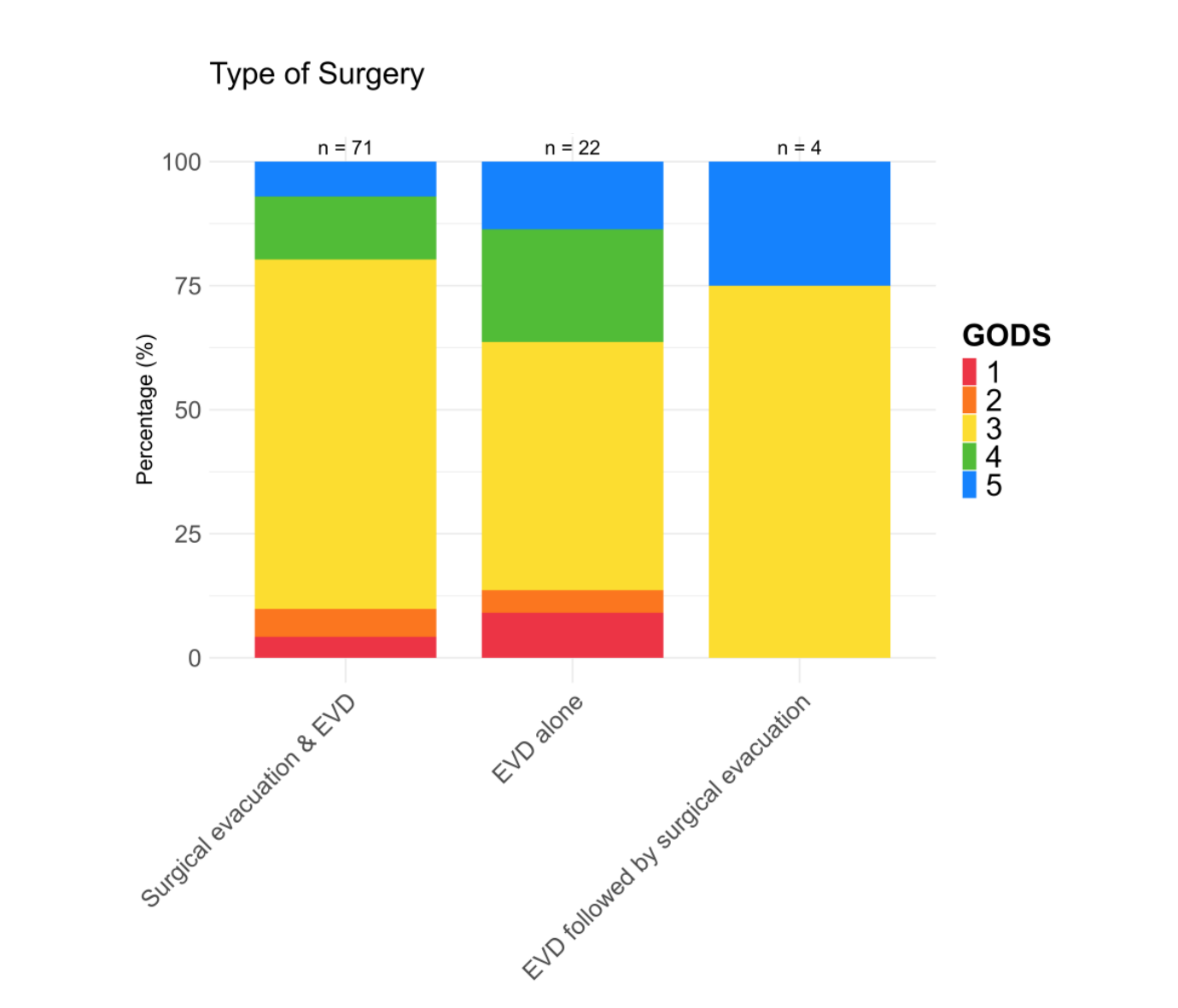


The figure illustrates the outcome distribution for each surgical approach.

EVD = External ventricular drainage. GODS = Glasgow Outcome Scale at Discharge.

**Supplementary Figure 3.** **Long-term mortality in relation to age in the surgical and conservatively managed sub-cohorts – Kaplan-Meier analyses**
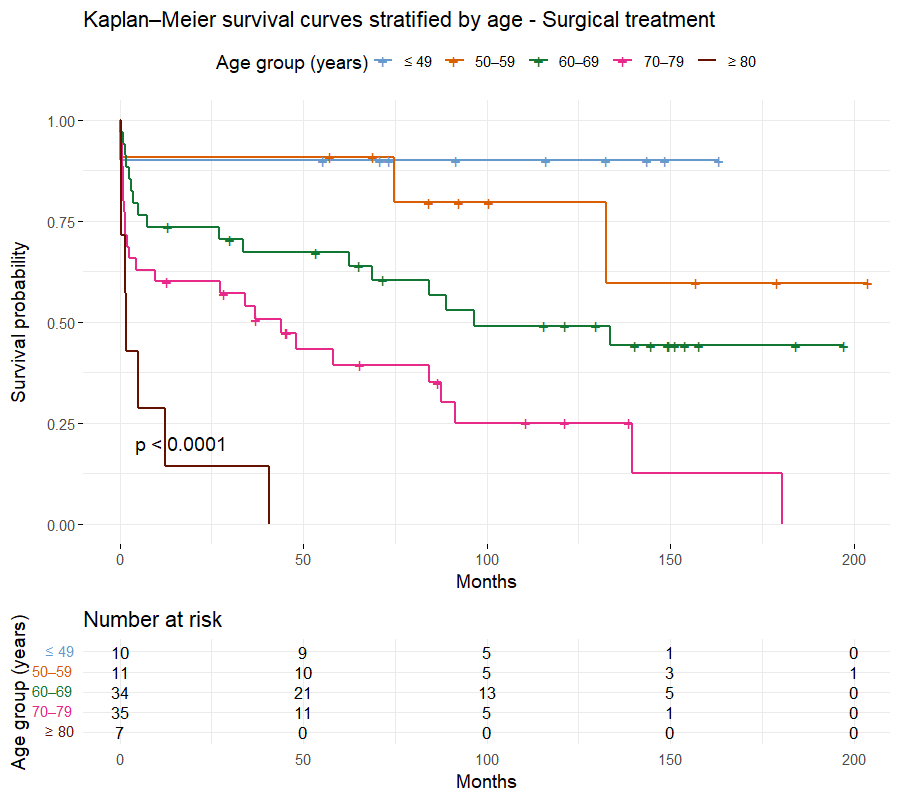


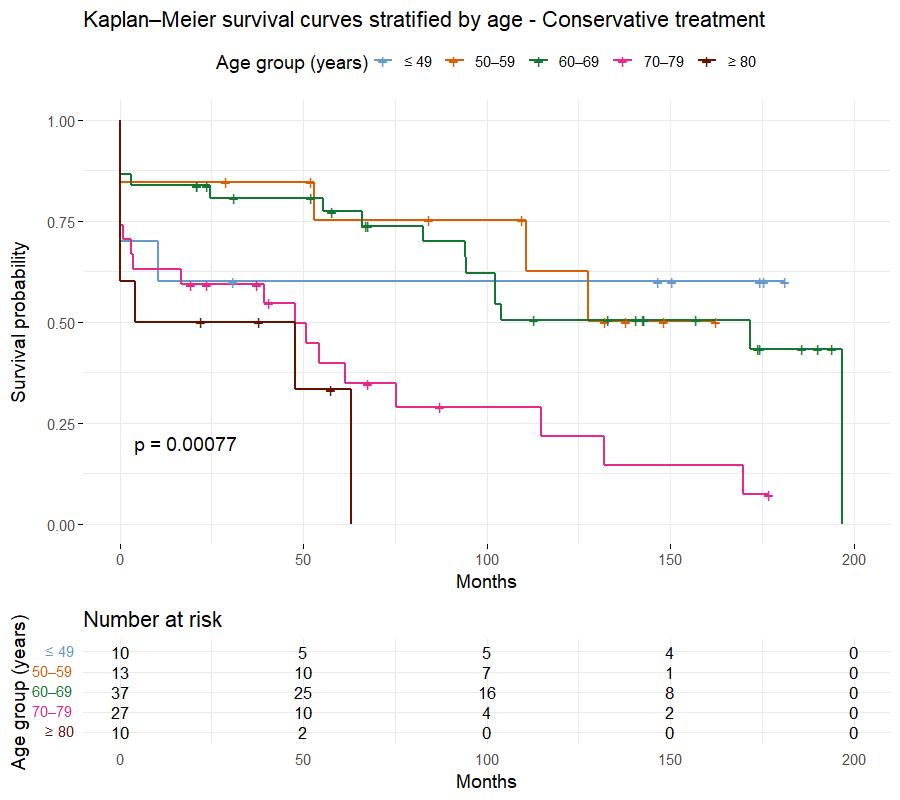


p < 0.05 indicates statistical significance.
