# Supplementary material for: Treatment strategies, complications, and outcomes in spontaneous cerebellar hemorrhage: a swedish observational single-center study
Source: Acta Neurochir (Wien). 2026 Apr 15;168(1):99. doi: 10.1007/s00701-026-06872-w (PMC13086794; doi:10.1007/s00701-026-06872-w)
Supplement: Supplementary file 2 — Supplementary Material 2 (DOCX 20.1 KB) [file 701_2026_6872_MOESM2_ESM.docx]

**Supplementary Table 1. Antithrombotic regime and reversal strategies**

| **Antithrombotic regime** | **Antithrombotic agent** | **Treatment strategy** | **Patients, n** | **Reversal** | **Patients, n (%)** |
| --- | --- | --- | --- | --- | --- |
| **Antiplatelet** | ASA | Conservative | 15 | Discontinuation | 15 (100%) |
|  |  | Surgery | 13 | Discontinuation | 8 (62%) |
|  |  |  |  | Discontinuation + Desmopressin | 2 (15%) |
|  |  |  |  | Discontinuation + Desmopressin + Platelet transfusion | 1 (8%) |
|  |  |  |  | Platelet transfusion + Desmopressin | 2 (15%) |
|  | Ticagrelor | Conservative | NA | NA | NA |
|  |  | Surgery | 1 | Discontinuation | 1 (100%) |
| **Anticoagulants** | VKA | Conservative | 10 | Discontinuation + VK PCC | 6 (60%) |
|  |  |  |  | Discontinuation + PCC | 3 (30%) |
|  |  |  |  | PCC | 1 (10%) |
|  |  | Surgery | 14 | Discontinuation + VK + PCC | 10 (71%) |
|  |  |  |  | Discontinuation + VK | 1 (7%) |
|  |  |  |  | Discontinuation + PCC | 3 (21%) |
|  | NOAC | Conservative | 4 | Discontinuation + PCC | 2 (50%) |
|  |  |  |  | Discontinuation + PCC + Tranexamic acid | 1 (25%) |
|  |  |  |  | Discontinuation + PCC + Other | 1 (25%) |
|  |  | Surgery | 6 | Discontinuation + VK + PCC | 1 (17%) |
|  |  |  |  | Discontinuation + PCC | 3 (50%) |
|  |  |  |  | Discontinuation + PCC + Tranexamic acid | 2 (33%) |
| **Antithrombotic combination** | Dual AP | Conservative | 3 | Discontinuation | 3 (100%) |
|  |  | Surgery | 1 | Platelet transfusion + Desmopressin | 1 (100%) |
|  | Dual AC | Conservative | 1 | Discontinuation + VK + PCC | 1 (100%) |
|  |  | Surgery | NA | NA | NA |
|  | AP + AC | Conservative | 7 | Discontinuation | 3 (43%) |
|  |  |  |  | Discontinuation + VK + PCC | 2 (29%) |
|  |  |  |  | Discontinuation + PCC | 2 (29%) |
|  |  | Surgery | 2 | Discontinuation | 1 (50%) |
|  |  |  |  | Discontinuation + PCC | 1 (50%) |

AC = Anticoagulant. AP = Antiplatelet. ASA = Acetylsalicylic acid. NA = Not applicable. NOAC = Novel oral anticoagulant. PCC = Prothrombin complex concentrate. VK = Vitamin K. VKA = VK antagonist.

**Supplementary Table 2. Clinical and radiological variables – in relation to surgical strategy**

|  | **Surgical cohort** | **Surgical evacuation + EVD** | **EVD alone** | **EVD -> Surgical evacuation** |
| --- | --- | --- | --- | --- |
| *Clinical variables* | | | | |
| GCS M (scale) before surgery, median (IQR) | 5 (4-6) | 5 (4-6) | 6 (4-6) | 6 (6-6) |
| Pupillary status before surgery (abnormal), n (%) | 6 (6%) | 2 (3%) | 4 (18%) | 0 (0%) |
| *Radiological variables* | | | | |
| Localization (uni-/bilateral), n (%) | 143/51 (74/26%) | 50/21 (70/30%) | 16/6 (73/27%) | 4/0 (100/0%) |
| Brainstem involvement (yes), n (%) | 12 (6%) | 9 (13%) | 1 (5%) | 1 (25%) |
| Hematoma volume (mL) before surgery, median (IQR) | 25 (16-38) | 29 (20-38) | 10 (6-17) | 23 (13-35) |
| Brainstem compression on first CT (none/minimal/moderate/severe), n (%) | 164/23/7/0 (85/12/4/0%) | 55/10/6/0 (78/14/9/0%) | 19/3/0/0 (86/14/0/0%) | 2/2/0/0 (50/50/0/0%) |
| Maximal brainstem compression (none/minimal/moderate/severe), n (%) | 159/24/11/0 (82/12/6/0%) | 54/11/6/0 (76/16/9/0%) | 18/3/1/0 (82/14/5/0%) | 1/3/0/0 (25/75/0/0%) |
| Brainstem compression (none/minimal/moderate/severe) before surgery, n (%) | 72/18/7/0 (74/19/7/0%) | 54/11/6/0 (76/16/9/0%) | 17/4/1/0 (77/18/5/0%) | 1/3/0/0 (25/75/0/0%) |
| Compression of the 4^th^ ventricle (none/minimal/moderate/severe) on first CT, n (%) | 54/81/50/9 (28/42/26/5%) | 7/35/25/4 (10/49/35/6%) | 6/11/4/1 (27/50/18/5%) | 1/1/2/0 (25/25/50/0%) |
| Maximal compression of the 4^th^ ventricle (none/minimal/moderate/severe), n (%) | 42/73/60/19 (22/38/31/10%) | 4/27/31/9 (6/38/44/13%) | 3/12/5/2 (14/55/23/9%) | 0/1/3/0 (0/25/75/0%) |
| Compression of the 4^th^ ventricle (none/minimal/moderate/severe) before surgery, n (%) | 6/42/39/10 (6/43/40/10%) | 4/27/32/8 (6/38/45/11%) | 2/14/4/2 (9/64/18/9%) | 0/1/3/0 (0/25/75/0%) |
| Tonsil herniation (yes) on first CT, n (%) | 15 (8%) | 10 (14%) | 0 (0%) | 0 (0%) |
| Maximal tonsil herniation (yes), n (%) | 20 (10%) | 10 (14%) | 3 (14%) | 0 (0%) |
| Tonsil herniation (yes) before surgery, n (%) | 13 (13%) | 10 (14%) | 3 (14%) | 0 (0%) |
| Graeb score (scale) on first CT, median (IQR) | 2 (0-4) | 2 (1-7) | 3 (1-6) | 2 (0-5) |
| Maximal Graeb score (scale), median (IQR) | 2 (0-5) | 2 (1-7) | 3 (2-6) | 3 (1-5) |
| Graeb score (scale) before surgery, median (IQR) | 3 (1-6) | 2 (1-7) | 3 (2-6) | 1 (1-3) |
| Evans’ index on first CT (ratio), median (IQR) | 0.30 (0.27-0.33) | 0.31 (0.27-0.34) | 0.30 (0.29-0.34) | 0.30 (0.28-0.33) |
| Maximal Evans’ index (ratio), median (IQR) | 0.32 (0.28-0.35) | 0.34 (0.30-0.37) | 0.32 (0.30-0.35) | 0.33 (0.32-0.34) |
| Evans’ index (ratio) before surgery, median (IQR) | 0.32 (0.28-0.34) | 0.32 (0.28-0.34) | 0.31 (0.29-0.35) | 0.30 (0.29-0.32) |

Abnormal pupillary status was defined as one or two unreactive pupils.

CT = Computed tomography. EVD = External ventricular drainage. GCS M = Glasgow Coma Scale Motor score. IQR = Interquartile range.
